# Prevalence and determinants of post-neonatal mortality in East Africa: a multilevel analysis of the recent demographic and health survey

**DOI:** 10.3389/fped.2025.1380913

**Published:** 2025-01-23

**Authors:** Alebachew Ferede Zegeye, Desale Bihonegn Asmamaw, Wubshet D. Negash, Tadele Biresaw Belachew, Elsa Awoke Fentie, Atitegeb Abera Kidie, Tsegaw Amare Baykeda, Samrawit Mihret Fetene, Banchlay Addis, Sisay Maru Wubante, Abel Endawkie, Tadesse Tarik Tamir

**Affiliations:** ^1^ Department of Medical Nursing, School of Nursing, College of Medicine and Health Sciences University of Gondar, Gondar, Ethiopia; ^2^ Department of Reproductive Health, Institute of Public Health, College of Medicine and Health Sciences, University of Gondar, Gondar, Ethiopia; ^3^ Department of Health Systems and Policy, Institute of Public Health, College of Medicine and Health Sciences, University of Gondar, Gondar, Ethiopia; ^4^ National Centre for Epidemiology and Population Health, The Australian National University, Canberra, ACT, Australia; ^5^ School of Public Health, College of Health Science, Woldia University, Woldia, Ethiopia; ^6^ Department of Health Informatics, Institute of Public Health, College of Medicine and Health Sciences, University of Gondar, Gondar, Ethiopia; ^7^ Department of Epidemiology and Biostatistics, School of Public Health, College of Medicine and Health Science, Wollo University, Wollo, Ethiopia; ^8^ Department of Pediatrics and Child Health Nursing, School of Nursing, College of Medicine and Health Sciences, University of Gondar, Gondar, Ethiopia

**Keywords:** determinants, East Africa, mortality, post-neonatal, prevalence

## Abstract

**Background:**

One of the most common measures of society's health is mortality among neonates. Developing and developed countries still differ significantly in neonatal mortality rates. While there are about 18 neonatal deaths worldwide for every 1,000 live births during the first month of life, less is known about neonatal mortality in developing countries, especially in East Africa. Understanding the extent of mortality during the post-neonatal period and its determinants is crucial for developing appropriate policies and strategies that could help solve the issue. Thus, the aim of this study was to identify the prevalence of post-neonatal mortality in East African countries and the factors that are associated with it.

**Methods:**

Secondary data analysis was conducted using data from the most recent Demographic and Health Surveys, which included 11 East African countries between 2014 and 2022. A weighted sample of 225,635 live births had been used in the study. STATA/SE 14 was used for data analysis. The multilevel mixed-effects logistic regression model was applied to determine the factors associated with post-neonatal mortality. In the multilevel logistic regression model, significant factors were deemed to be associated with post-neonatal mortality at *p*-values <0.05. The data were interpreted using the adjusted odds ratio (AOR) and confidence interval (CI). The best-fit model has been found to be the one with the lowest deviance and highest logliklihood ratio.

**Results:**

In East Africa, post-neonatal mortality was found to be 15 per 1,000 live births. Pregnancy type (AOR = 3.09, 95% CI: 2.30, 4.13), birth weight (AOR = 1.58, 95% CI: 1.25, 2.01), maternal age (AOR = 1.58, 95% CI: 1.32, 1.90), maternal education (AOR = 1.82, 95% CI: 1.14, 2.92), tetanus shots prior to delivery (AOR = 1.23; 95% CI: 1.06–1.42), birth order (AOR = 5.68, 95% CI: 4.48, 7.24), those born in Uganda (AOR = 1.33, 95% CI: 1.03, 1.73), and Burundi (AOR = 1.48, 95% CI: 1.11, 1.98) had the highest odds of post-neonatal death.

**Conclusion:**

According to this study, post-neonatal mortality is higher in developing countries, particularly in East Africa. It was discovered that factors at the individual and community levels associated with post-neonatal mortality. Consequently, focus should be paid to babies born to mothers in the lowest age group, those born of multiple pregnancies, without formal educations, who did not receive tetanus shots prior to birth, and who were born in the first birth order.

## Introduction

The number of infant deaths between the ages of 28 days and 11 months is known as post-neonatal mortality, and it is determined by dividing the total number of post-neonatal deaths by the number of live births in a particular year (1). Infant mortality is a good and affordable way to measure population health. The health of the child and society at large are both indicated by neonatal mortality (2). The high postnatal mortality rate is a result of the predominance of unfavorable social, economic, and environmental situations during the first year of life (3). Because they have considerably weaker immune systems than adults, newborns are far more susceptible to challenges in the environment and in community. They also require assistance because they are unable to care for themselves. Infants typically suffer the most from poor living conditions as a result (4).

Worldwide, there is a huge disparity in the post-neonatal death rate. Diarrhea, acute respiratory illness, measles, tetanus, and malaria are among the diseases that can be readily treated or prevented and are the main reasons of post-neonatal mortality. In developing countries, a high and significantly variable proportion of infants still die each year from these and other reasons (5–7). A child's first month of existence carries the biggest risk of death; in 2021, the average global rate of deaths per 1,000 live births was 18 (down 51% from 37 in 1990). In contrast, it was calculated that there were 11 deaths for every 1,000 people who lived past the first month of life and before turning 1 year (8, 9). Only a small decline in postnatal deaths is seen globally each year. However, a number of developing countries, such as those in Eastern Africa, are still far left behind (10).

The risk of post-neonatal death is 55 per 1,000 live births in African countries, which is more than five times higher than the rate of 10 per 1,000 live births in European countries (11, 12). Post-neonatal mortality in Sub-Saharan Africa, particularly in East Africa, has increased gradually as a result of the region's ongoing high rates of pneumonia, diarrhea, malaria, and vaccine-preventable infections (13, 14).

Research conducted globally has shown a substantial association between post-neonatal mortality with place of delivery (15), means of delivery (16), number of antenatal visits (17), birth interval (18), educational status of the mother (19–21), type of place of residence (15), distance to health institution (22, 23), birth order number (24), pregnancy status (25), child weight at birth (26), type of pregnancy (27), and child sex (28).

To the best of our knowledge and literature search, no research has been done on the post-neonatal mortality rate in East Africa, despite the fact that those countries bear a significant portion of the world's infant mortality burden. Thus, the current study uses multilevel mixed effect analysis of the most recent Demographic and Health Survey data to investigate the prevalence and factors associated with post-neonatal mortality in East Africa.

## Methods and materials

### Study design and period

Through secondary analysis, a population-based cross-sectional study was carried out using data from the Demographic and Health Survey (DHS) of 11 East African countries between 2014 and 2022. To generate updated health and health-related indicators, a community-based cross-sectional Demographic and health survey is conducted every 5 years.

### Data source, study population and sampling technique

Based on the most recent East African countries Demographic Health Surveys (DHS) datasets from 2014 to 2022, a secondary data analysis was carried out. The DHS surveys from eleven East African countries such as Burundi, Ethiopia, Comoros, Uganda, Rwanda, Tanzania, Mozambique, Zimbabwe, Kenya, Zambia, and Malawi were employed in our analysis. To determine the prevalence and factors associated with post-neonatal mortality in Eastern Africa, the data were appended together. Every country's survey contains a variety of datasets, including those related to men, women, children, births, and households. Using a stratified two-stage cluster technique, DHS selects enumeration areas (first stage) and then draws a sample of households from each selected enumeration area (second stage). The age at death (b7) variable was recoded from the kid's Record (KR) dataset in order to calculate the outcome variable (post-neonatal mortality).

The factors associated with post-neonatal mortality were determined using a binary logistic regression model. In the bivariable analysis, unadjusted odds ratios (ORs) with a 95% confidence interval were calculated to identify potential candidate variables for the multivariable analysis. Variables with *p*-values less than 0.25 in the bivariable analysis were considered suitable for inclusion in the multivariable analysis. In the multivariable analysis, adjusted odds ratios (AORs) with a 95% confidence interval were reported to account for potential confounders, and variables with *p*-values less than 0.05 were considered statistically significant. We used the weighting variable (v005) as a relative weight normalized to make the analysis survey-specific. For the pooled data, we denormalized the post-neonates’ individual standard weight variable by dividing it by the sampling fraction of each country. The post-neonates’ adjusted weight was calculated as follows: Post-neonates’ adjusted weight = V005 × (number of post-neonates aged 28 days to 11 months in the country at the time of the survey)/(total number of post-neonates aged 28 days to 11 months interviewed in the survey). In all, 225,635 live births were included in the weighted sample for this study (Table 1).

**Table 1:** Sample size for post-neonatal mortality and its determinants among post-neonates in East Africa, DHS 2014–2022.

Countries	Year of survey	Weighted frequency	Weighted percent
Burundi	2016/17	13,187	5.84
Ethiopia	2016	20,922	9.27
Kenya	2022	41,810	18.53
Comoros	2014	5,604	2.48
Malawi	2015/16	34,414	15.25
Mozambique	2015	10,328	4.58
Rwanda	2019/20	16,150	7.16
Tanzania	2022	20,394	9.04
Uganda	2016	30,994	13.74
Zambia	2018	19,746	8.75
Zimbabwe	2015	12,086	5.36
Total sample size		225,635	100

### Study variables

#### Dependent variables

Post-neonatal death in months was the study's outcome variable. The number of infant deaths between the ages of 28 days and 11 months is known as post-neonatal mortality, and it is measured as the number of neonatal deaths per 1,000 live births in a year. Neonatal deaths were dichotomized into “yes = 1” for those who passed away between the ages of 28 days and 11 months and “no = 0” for those who lived (29).

#### Independent variables

**Figure 1:**
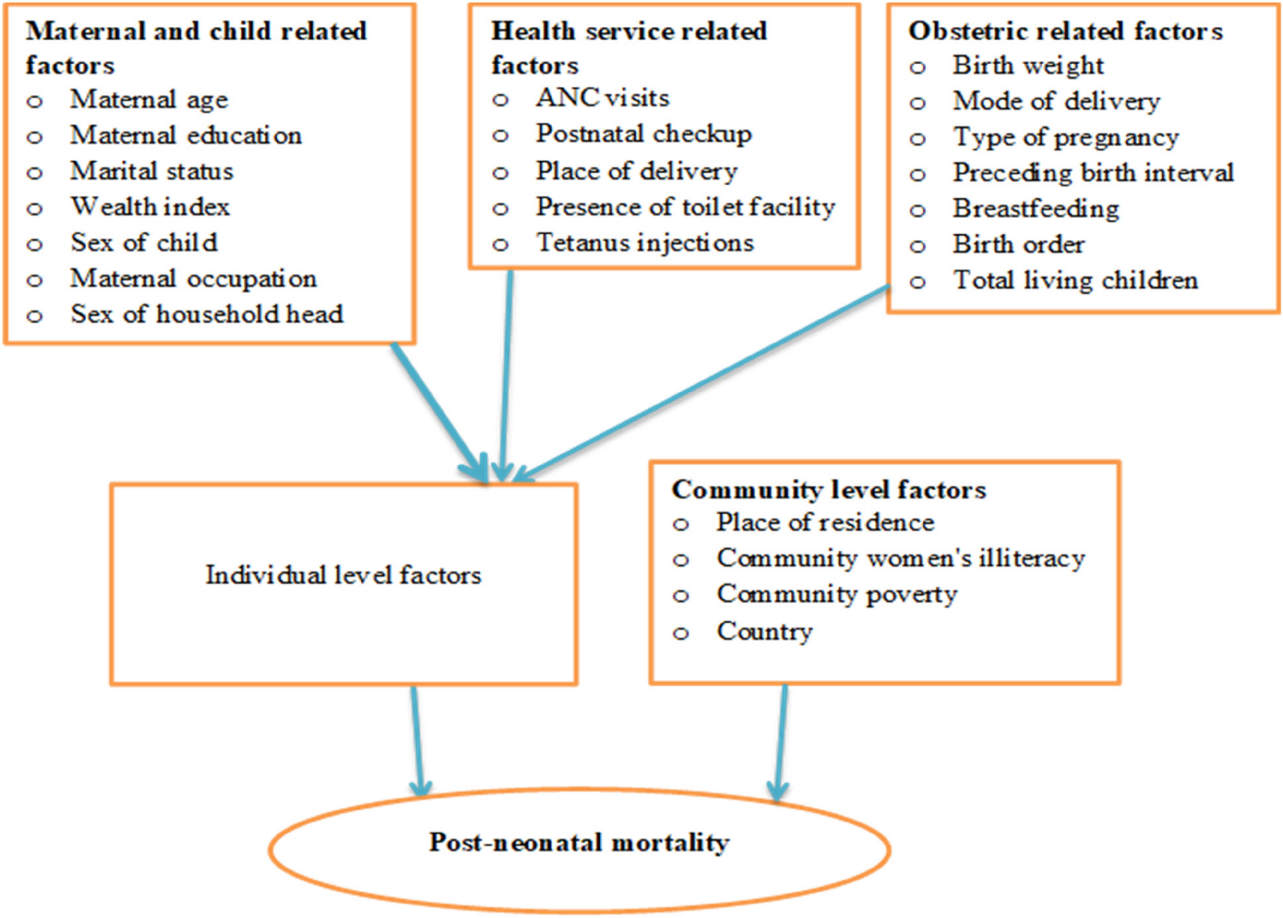
Conceptual framework for variables associated with post-neonatal mortality in East Africa.

The independent factors taken into consideration for this study was obtained from two sources (individual-level and community-level variables), due to the hierarchical nature of DHS data. Maternal age (15–24, 25–34, 35–49), maternal education (no formal education, primary, secondary, or higher), and maternal occupation (has no occupation, has occupation), the mother's marital status (single, married, other), wealth index (poor, middle, rich), child's sex (male, female), sex of the head of the household (male, female), Birth weight (Normal, High, or Low), delivery location (home, health facility), delivery mode (CS) (Yes, No), pregnancy type (single, multiple), birth interval (≤24, >24) breast feeding duration (ever breastfed, not currently breastfeeding, never breastfed, still breastfeed, others), give a youngster something besides breast milk (Yes, No). The number of ANC visits (<4, ≥4), shots of tetanus prior to birth (Yes, No), Ever received a vaccination (Yes, No), birth order (First-order, 2–4, greater than 4), the number of children still alive (less than or equal to three, more than three), Presence of a toilet (Yes, No). The community-level factors were country of residence (Burundi, Ethiopia, Kenya, Comoros, Malawi, Mozambique, Rwanda, Tanzania, Uganda, Zambia, Zimbabwe), place of residence (Urban, Rural), community women's illiteracy (Low, High), and community poverty (Low, High) (Figure 1; Table 2).

**Table 2:** Summary of model name, objective, independent variables, dependent variable, and expression of result.

Model name	Objective	Independent variables	Dependent variable	Expression of result
Null Model (Model 0)	To investigate the variation in post-neonatal death rates within clusters without including exposure variables.	None (Random intercept-only model)	Post-neonatal mortality	ICC, MOR, and PCV were used to measure variation across clusters
Model I	To assess the association between individual-level factors and post-neonatal mortality	Maternal age, education, occupation, marital status, wealth index, child sex, head of household sex, birth weight, delivery location, delivery mode, pregnancy type, birth interval, breastfeeding duration, number of ANC visits, vaccination	Post-neonatal mortality	Adjusted Odds Ratio (AOR) and 95% Confidence Intervals (CI)
Model II	To assess the association between community-level factors and post-neonatal mortality	Country of residence, place of residence, community-level women illiteracy, community-level poverty	Post-neonatal mortality	AOR and 95% CI
Model III	To evaluate the combined association of individual-level and community-level factors with post-neonatal mortality.	All variables from Model I and Model II	Post-neonatal mortality	AOR and 95% CI, with measures of random effects (ICC, MOR, PCV).
Best-Fit Model	To identify the most appropriate model for explaining post-neonatal mortality by comparing log-likelihood and deviance values	Variables from Model I and Model II contributing to the best fit	Post-neonatal mortality	The model with the lowest deviance and highest log-likelihood ratio was chosen

### Description of independent variables

#### Wealth index

The wealth index is a composite indicator of a household's total standard of living. The wealth index is created using conveniently collected data on the items that a household owns, like televisions and bicycles, the materials used to build a home, the types of water access, and the availability of sanitary facilities (30).

#### Community-level women illiteracy

Data on respondents’ educational attainment is used to calculate the percentage of women with at least a primary education. Following the calculation of cross-tabulating the individual level of women's education with the cluster number (v001), it was then classified using the national mean value: low community level women's illiteracy (communities with ≥50% of the national mean value of women's education) and high community-level women's illiteracy (communities with <50% of the national mean value of women's education).

#### Community-level poverty

It is produced by taking into account the percentage of women in the rich and middle-class categories. It was then categorized using the national mean value of the wealth index following the computation of the cross-tabulating individual-level combined wealth index with the cluster number (v001): low community-level poverty (communities with ≥50% of the national mean value of the community wealth index) and high community-level poverty (communities with <50% of the national mean value of the community wealth index).

### Data management and statistical analysis

The data extracted from recent DHS data sets were cleaned, entered, and analyzed with the statistical software STATA/SE version 14. The DHS data contains nested clusters of variables, and the similarities between variables inside a cluster are greater than those between variables outside of it. To use a standard logistic regression model, the assumptions of independent data and equal variance across clusters were broken. This suggests that an advanced model must be used to account for between-cluster variations (31).

In light of this, post-neonatal mortality was determined by using multilevel mixed-effects logistic regression to identify the associated factors. Four models are used in multilevel mixed effect logistic regression: model I (which only includes individual level variables), model II (which only includes community level factors), and model III (which includes both individual and community level variables) (32). The null model, which does not include exposure factors, was employed to investigate the variation in post-neonatal death rates within the cluster.

The association between community-level and individual-level variables and the outcome variable (Model II) and Model I, respectively, was evaluated. The association between the individual and community-level factors and the outcome variable (post-neonatal mortality) was fitted simultaneously in the final model, or Model III. The models were compared using the deviance and log-likelihood tests; the model with the highest log-likelihood ratio and the lowest deviance was found to be the best-fitting one. Additionally, the variance inflation factor (VIF) was used to test for multicollinearity. In this analysis, missing data were addressed using the STATA command “drop if variable ==.” which ensures the exclusion of observations with missing values for the specified variable(s) from the analysis. The results show that there was no significant multicollinearity across the independent variables, with a mean VIF of 1.74 and a VIF of less than five for each independent variable.

### Random effects

The random effects or measures of variation of the outcome variables were measured using the intra-class correlation coefficient (ICC), proportional change in variance (PCV), and median odds ratio (MOR). A proportional change in variance (PCV) and intra-class correlation coefficient (ICC) were calculated to determine the difference between the clusters. Using clusters as a random variable, the ICC indicates that the difference in post-neonatal death rates across clusters can be calculated as follows: $\;\mathrm{ICC}=\frac{\mathrm{VC}}{\mathrm{VC}+3.29}\times 100\text{\%}$. When two clusters are randomly selected, using clusters as a random variable, the MOR is the median value of the odds ratio between the area with the highest risk and the area of the lowest risk for post-neonatal mortality: MOR =  *e*^0.95√VC^.

Additionally, the PCV shows how variables account for the variation in the prevalence of post-neonatal mortality, which is calculated as: $\;\mathrm{PCV}=\frac{V\mathrm{null}-VC}{V\mathrm{null}}\times 00\text{\%}$; where VC is the cluster level variance and *V*null is the variance of the null model (32–34). The likelihood of post-neonatal mortality was estimated using random effects and independent variables at the individual and community levels. With a *p*-value of less than 0.05, the adjusted odds ratio (AOR) and 95% confidence intervals were used to judge it and show its strength. Because the data set is nested, a deviance = −2 log likelihood ratio was used to compare the models; the model with the lowest deviance was chosen as the best-fit model. By calculating the variance inflation factors (VIF), the multi-collinearity of the variables employed in the models was confirmed, and the results were found to be within reasonable bounds of one to ten.

## Results

### Socio-demographic and economic characteristics of post-neonates in East Africa

The analysis comprised 225,641 live births, of which 113,708 were male and 111,933 were female. The majority of the babies (32.16%) were born to moms who were unemployed. 73,468 (45.41%) babies were born whose mothers attended less than four antenatal care visits during their pregnancy, and more than one-third (75.61%) of the participants were born to mothers who lived in rural areas of east Africa. Over half (54.91%) of the babies were delivered to mothers who didn't have a high degree of literacy in their community (Table 3).

**Table 3:** Socio-demographic and economic characteristics of respondents in east Africa, DHS 2014–2022.

Variables	Frequency	Percent
Individual level variables		
Maternal age		
15–24	67,814	30.05
25–34	106,088	47.02
35–49	51,739	22.93
Maternal educational status		
No formal education	50,597	22.42
Primary	118,600	52.56
Secondary	47,833	21.20
Higher	8,611	3.82
Maternal occupation		
Has no occupation	65,522	32.16
Has occupation	138,219	67.84
Marital status of the mother		
Single	11,633	5.16
Married	191,570	84.90
Other	22,438	9.94
Wealth index		
Poor	107,539	47.66
Middle	40,924	18.14
Rich	77,178	34.20
Sex of child		
Male	113,708	50.39
Female	111,933	49.61
Sex of household head		
Male	168,973	74.89
Female	56,668	25.11
Birth weight		
Low	72,305	37.37
Normal	101,418	52.42
High	19,754	10.21
Place of delivery		
Home	61,081	27.07
Facility	164,560	72.93
Mode of delivery CS		
Yes	201,025	93.53
No	13,907	6.47
Type of pregnancy		
Single	218,381	96.78
Multiple	7,260	3.22
Preceding birth interval (months)		
≤24	36,133	21.40
>24	132,740	78.60
Breastfeeding duration		
Ever breast breastfeed	105,543	53.03
Never breastfeed	6,421	3.23
Still breastfeeding	72,757	36.55
Others	14,320	7.19
Give child anything other than breast milk		
No	104,951	89.22
Yes	12,682	10.78
Number of antenatal visits during pregnancy		
<4	73,468	45.41
≥4	88,309	54.59
Tetanus injections before birth		
Yes	107,767	77.96
No	30,466	22.04
Child ever had vaccinated		
No	7,559	17.20
Yes	36,394	82.80
Birth order number		
First-order	52,697	23.35
2–4	109,235	48.41
Total number of living children		
1	63,709	28.23
≤3	131,782	58.40
≥4	93,859	41.60
Presence of toilet facility		
No	35,979	15.95
Yes	189,662	84.05
Community level variables		
Type of place of residence		
Urban	55,043	24.39
Rural	170,598	75.61
Community-level women's illiteracy		
Low	123,895	54.91
High	101,746	45.09
Community-level wealth index		
Low	115,736	51.29
High	109,905	48.71
Country of residence		
Burundi	13,187	5.84
Ethiopia	20,922	9.27
Kenya	41,810	18.53
Comoros	5,610	2.49
Malawi	34,414	15.25
Mozambique	10,328	4.58
Rwanda	16,150	7.16
Tanzania	20,394	9.04
Uganda	30,994	13.74
Zambia	19,746	8.75
Zimbabwe	12,086	5.36

### Prevalence of post-neonatal mortality among post-neonates in east African countries

**Figure 2:**
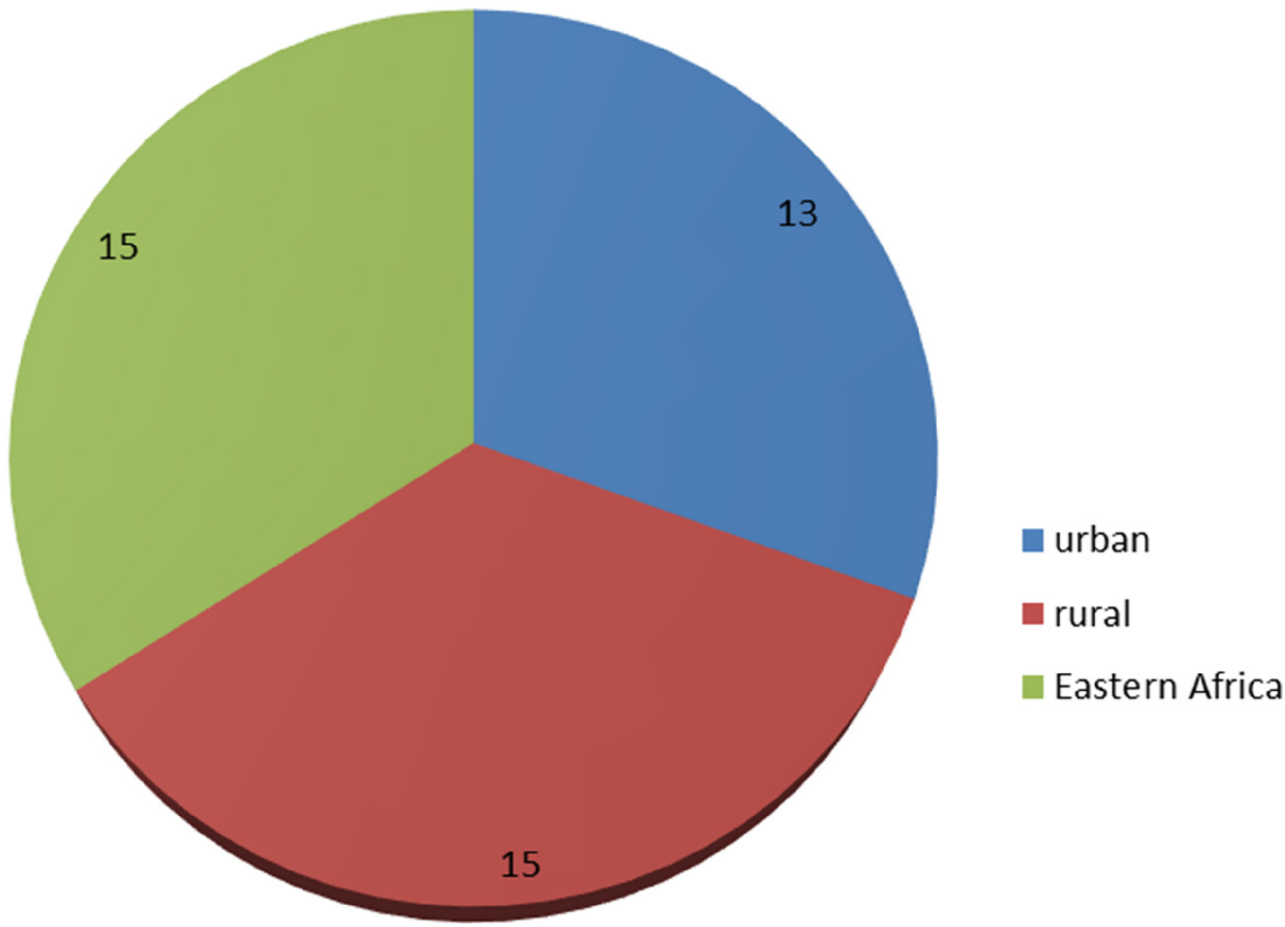
Prevalence of post-neonatal mortality in East Africa per 1,000 live births.

**Figure 3:**
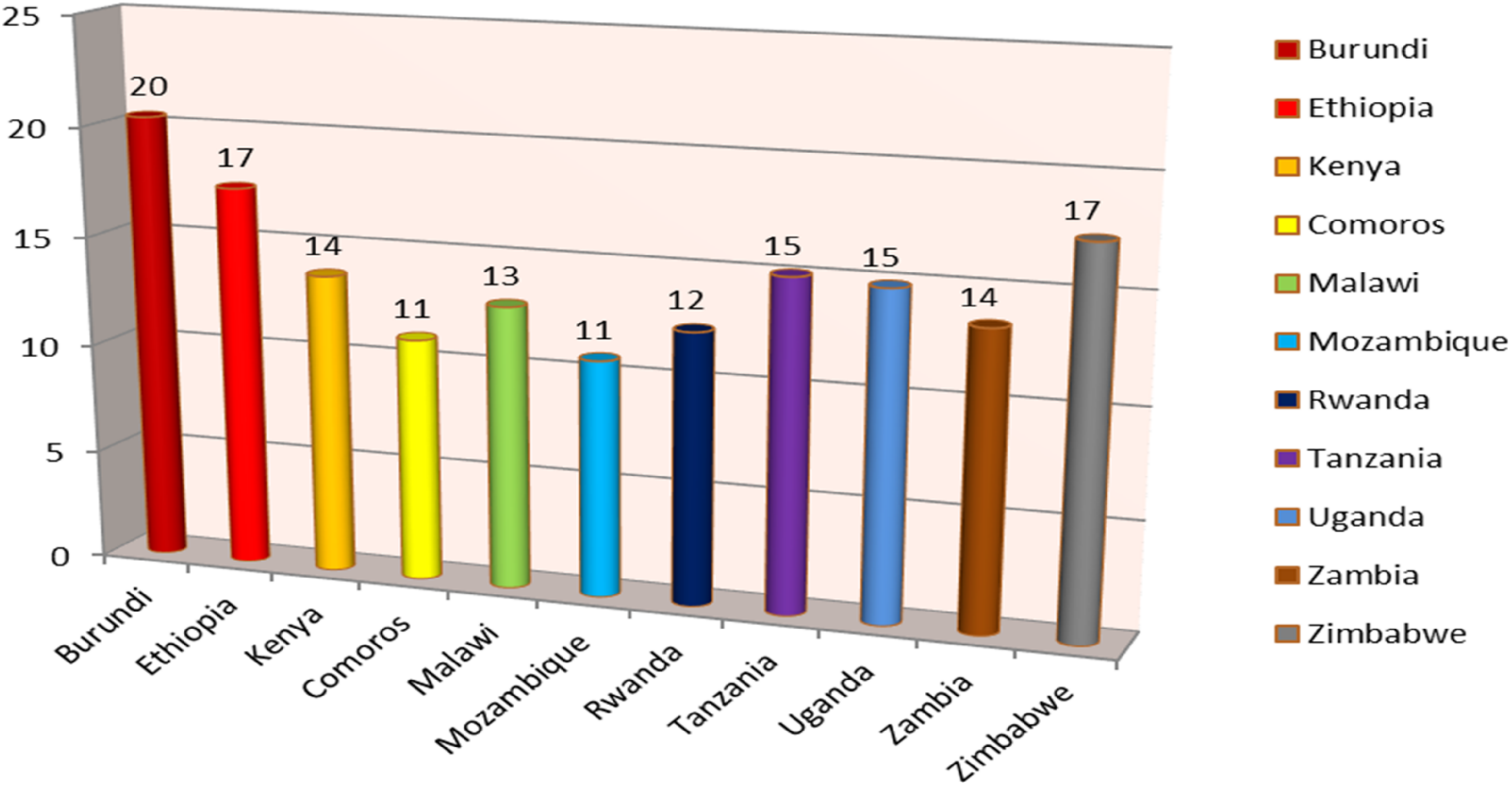
Regional prevalence of post-neonatal mortality in East Africa per 1,000 live births.

In East Africa, the prevalence of post-neonatal mortality was 15 per 1,000 live births. In east Africa, the prevalence of post-neonatal mortality has been found to be 13 and 15 deaths per 1,000 live births, respectively, in urban and rural areas (Figure 2). In East Africa, Burundi had the greatest rate of post-neonatal mortality (20 deaths per 1,000 live births) while Comoros had the lowest rate (11 deaths per 1,000 live births) (Figure 3).

### Random effect and model fitness

In order to test whether the information supported the choice to evaluate randomness at the community level, a null model was conducted. The null model's findings demonstrated that the post-neonatal death rate differed greatly between communities, with variance = 0.7788531 and a *p* value of <0.001. According to the null model's ICC value, variance across clusters accounted for 19.14% of the variation in post-neonatal mortality, whereas within-cluster variation accounted for 80.86% of the variation. The probabilities of post-neonatal mortality in the null model were 2.31 times different between higher and lower risk clusters. According to Model I's intraclass correlation value, 16.65% of the variations in post-neonatal mortality were accountable for the variations between communities. Then we built Model II using only community-level variables in the null model; based on the ICC value from Model II, cluster distinctions explained 12.92% of the variation in post-neonatal mortality. In the final model (model III), the risks of post-neonatal mortality varied 1.63 times across low and high risk clusters. This model attributed approximately 66.29% of the variation in odds of post-neonatal mortality to both individual and community-level determinants (PCV = 66.29%) (Table 4).

**Table 4:** Model comparison and random effect analysis post-neonatal mortality in east Africa.

Parameter	Null model	Model I	Model II	Model III
Variance	0. 7788531	0.657086	0.4880574	0.2625732
ICC	19.14%	16.65%	12.92%	7.39%
MOR	2.31	2.16	1.94	1.63
PCV	Reference	15.63%	37.34%	66.29%
Model fitness				
LLR	−16,914.838	−5,976.1991	−16,872.951	−5,961.1076
Deviance	33,829.676	11,952.3982	33,745.902	11,922.2152

ICC, interacluster correlation; MOR, median odds ratio; PCV, proportional change in variance; LLR: logliklihood ratio.

### Factors associated with post-neonatal mortality in East Africa

Maternal age (15–24), maternal education (no formal education), low birth weight, type of pregnancy (multiple), not receiving tetanus shots prior to birth, birth order (>4), number of living children (>3), and countries (Burundi and Uganda) were significantly associated with post-neonatal mortality in the best-fit model of multivariable multilevel logistic regression at a *p*-value of <0.001.

Children born to mothers between the ages of 15 and 24 had a 1.58 higher risk of post-neonatal death than children born to mothers between the ages of 25 and 34 (AOR = 1.58, 95% CI: 1.32, 1.90). The odds of post-neonatal death were 1.82 times greater for babies born to mothers with no formal education (AOR = 1.82, 95% CI: 1.14, 2.92) than for babies born to mothers with higher levels of education. Low birth weight babies had a 1.59 times greater chance of dying between the ages of 1 and 11 months compared to children born at a normal birth weight (AOR = 1.58, 95% CI: 1.25, 2.01). The odds of post-neonatal death were 3.09 times higher for post-neonates born of multiple pregnancies than for those born of a single pregnancy (AOR = 3.09, 95%.

Compared to newborns delivered to women who had tetanus shots before to delivery, the odds of post-neonatal mortality were 1.23 times higher for babies born to mothers who did not receive tetanus injections (AOR = 1.23; 95% CI: 1.06–1.42). Compared to babies in the 2–4 birth order, those in the first birth order had a 5.68 higher chance of dying between the ages of 1 and 11 months (AOR = 5.68, 95% CI: 4.48, 7.24). Children born to mothers in Burundi (AOR = 1.48, 95% CI: 1.11, 1.98) and Uganda (AOR = 1.33, 95% CI: 1.03, 1.73) had 1.48 and 1.33 times a higher risk of post-neonatal mortality, respectively, than babies born to mothers in Ethiopia (Table 5).

**Table 5:** Multivariable multilevel logistic regression analysis of individual-level and community level factors associated with post-neonatal mortality in east Africa, DHS 2014–2022.

Variables	Model I AOR (95% CI)	Model II AOR (95% CI)	Model III AOR (95% CI)
Maternal age			
15–24	1.55 (1.20, 1.84)		**1.58 (1.32, 1.90)**
25–34	1		1
35–49	0.97 (0.82, 1.15)		0.94 (0.79, 1.11)
Maternal education			
No formal education	1.69 (1.07, 2.68)		**1.82 (1.14, 2.92)**
Primary	1.71 (1.10, 2.66)		1.85 (0.18, 2.88)
Secondary	1.33 (0.86, 2.08)		1.35 (0.86, 2.10)
Higher	1		1
Maternal occupation			
No	0.81 (0.71, 0.94)		0.83 (0.71, 1.96)
Yes	1		1
Marital status of the mother			
Single	1.35 (1.05, 1.73)		1.34 (0.04, 1.72)
Married	1		1
Other	1.36 (1.12, 1.65)		1.36 (0.13, 1.65)
Wealth index combined			
Poor	1.13 (0.97, 1.33)		1.09 (0.91, 1.30)
Middle	0.98 (0.82, 1.19)		0.96 (0.79, 1.17)
Rich	1		1
Sex of child			
Male	1.22 (1.08, 1.37)		1.22 (0.08, 1.37)
Female	1		1
Sex of household head			
Male	1.2 (0.23, 7.40)		0.94 (0.81, 1.10)
Female	1		1
Birth weight			
Low	1.60 (1.27, 2.03)		**1.59 (1.25, 2.01)**
Normal	1		1
High	0.94 (0.76, 1.17)		0.93 (0.75, 1.16)
Place of delivery			
Home	0.93 (0.78, 1.11)		0.96 (0.80, 1.15)
Facility	1		1
Mode of delivery CS			
Yes	0.82 (0.63, 1.06)		0.85 (0.65, 1.11)
No	1		1
Type of pregnancy			
Single	1		1
Multiple	3.03 (2.27, 4.06)		**3.09 (2.30, 4.13)**
Give a child anything other than breast milk			
Yes	0.97 (0.81, 1.17)		1.01 (0.83, 1.23)
No	1		1
ANC visits during pregnancy			
<4	1.05 (0.93, 1.19)		1.10 (0.97, 1.24)
≥4	1		1
Tetanus injections before birth			
Yes	1		1
No	1.23 (1.06, 1.42)		**1.23 (1.06, 1.42)**
Birth order			
First-order	5.85 (4.60, 7.45)		**5.68 (4.48, 7.24)**
2–4	1		1
>4	1.16 (0.97, 1.39)		1.20 (0.01, 1.44)
Number of living children			
≤3	1.48 (1.26, 1.31)		1.12 (0.38, 1.53)
>3	1		1
Toilet facility			
No	1.14 (0.95, 1.37)		1.18 (0.98, 1.43)
Yes	1		1
Community level variables			
Place of residence			
Urban		1	1
Rural		1.12 (1.02, 1.23)	0.99 (0.82, 1.21)
Community-level women's illiteracy			
Low		0.99 (0.88, 1.12)	1.00 (0.84, 1.20)
High		1	1
Community-level poverty			
Low		0.96 (0.85, 1.084)	0.89 (0.75, 1.06)
High		1	1
Country of residence			
Burundi		1.16 (1.98, 1.36)	**1.48 (1.11, 1.98)**
Ethiopia		1	1
Kenya		0.75 (0.66, 0.88)	0.81 (0.53, 1.43)
Comoros		0.66 (0.50, 0.87)	0.67 (0.41, 1.86)
Malawi		0.73 (0.63, 0.84)	1.05 (0.81, 1.37)
Mozambique		0.64 (0.51, 0.80)	0.69 (0.54, 1.54)
Rwanda		0.71 (0.60, 0.85)	0.89 (0.65, 1.23)
Tanzania		0.87 (0.74, 1.01)	0.93 (0.70, 1.23)
Uganda		1.86 (1.75, 1.99)	**1.33 (1.03, 1.73)**
Zambia		0.78 (0.66, 0.92)	1.30 (0.98, 1.72)
Zimbabwe		1.03 (0.86, 1.23)	0.13 (0.01, 1.88)

ANC, antenatal care; CS, caesarean section. Model III: is the best-fit model since it has the highest log-likelihood ratio and the lowest deviance.

Bold indicates values that are statistically significant.

## Discussion

In developing countries like East Africa, post-neonatal deaths play a significant role in increasing childhood mortality. The purpose of this study was to determine the prevalence and determinants of post-neonatal mortality in East Africa. In East Africa, the prevalence of post-neonatal mortality was found to be 15 post-neonatal mortality per 1,000 live births. The finding is higher than the previous studies conducted in north African countries such as South Africa (35) and Gambia (36). The higher prevalence of post-neonatal mortality in this study than previous findings in South Africa and Gambia could be due to differences in socio-economic status and variability in health infrastructure and health policy.

The finding is lower than the previous studies conducted in African countries, such as Ethiopia (29), Kenya (37), and Tanzania (38). These variations might be due to differences in aggregate data and individual data. We used appended or aggregated data from individual countries that is averaged by geographic area and year. So individual data are disaggregated results which show the highest individual results compared to appended data.

In the multivariable logistic regression; Maternal age (15–24), maternal education (No formal education), low birth weight, type of pregnancy (multiple), not receiving tetanus shots prior to birth, Birth order (first birth order), Burundi, and Uganda were shown to be strongly associated with post-neonatal mortality in East Africa.

A significant predictor of post-neonatal death in this study was the mother's age. The odds of post-neonatal mortality were 1.58 times higher among babies born to mothers aged 15 and 24 years compared to babies born to mothers in age groups between 25 and 34. This finding is consistent with earlier findings (29, 39). The association between post-neonatal mortality and belonging to the 15–24 year old maternal age group could perhaps stem that the woman was not yet fully developed physically or physiologically for pregnancy. Along with physical or physiological immaturity, another factor can be related to a lack of prior childcare experience. Furthermore, post-neonatal deaths are more frequent in younger mothers because they are more likely to have preterm deliveries, low birth weight babies, and babies with congenital abnormalities (40, 41).

Compared to newborns born to mothers with greater levels of education, the risks of post-neonatal mortality were 1.82 times higher for babies born to mothers with no formal education. The results of earlier studies corroborate the conclusions of this finding (42, 43). This could be a feasible explanation for why raising maternal education levels is one of the most crucial steps to enhancing not just maternal and child health but also household production and the mother's and family's socio-emotional status (44). Achieving good maternal education will enhance women's socioeconomic status and health outcomes, which will have a favorable impact on child survival.

In relation to birth weight, Low-birth-weight babies had a 1.59 times greater chance of passing away between the ages of 1 and 11 months than babies born at a normal weight. This result is consistent with prior investigations (43, 45). Prematurity, intrauterine growth restriction, or both might result in low birth weight. Thus, one explanation for low birth weight children could be because they have immature organs and medical illnesses such as down syndrome, congenital heart disease, and diabetes mellitus (DM), which could raise their risk of death in the post-neonatal period (46).

The odds of post-neonatal mortality were 3.09 times higher among multiple pregnancies compared with singleton pregnancies. This finding is supported by the study findings (47, 48). This might be a logical explanation for why newborns from multiple pregnancies typically have restricted growth, poor Apgar scores, and extremely low birth weights. Furthermore, complications during pregnancy, labor, and postpartum are more likely in multiple pregnancies. In addition, due to increased food consumption, multiple pregnancies result in lower weight competition (49). The odds of post-neonatal mortality were 1.23 times higher among babies born to mothers who did not receive tetanus injections compared babies delivered to mothers who received tetanus shots before birth. The outcome of this study is in line with findings (50, 51). A possible explanation for this is that the tetanus vaccine creates antibodies that are protective against post-neonatal tetanus.

First-born babies had a 5.68 times higher risk of dying between the ages of 1 and 11 months as compared to children in birth orders two to four. This finding is consistent with (29, 52, 53). One explanation for this could be that babies born as firstborns are more vulnerable to pregnancy-related problems such Antepartum Hemorrhage (APH), preeclampsia, preterm, and fetal distress, which can raise their chance of dying between the ages of 1–11 months (54).

Furthermore, statistically, geographical regions (countries in East Africa) were associated with post-neonatal mortality. The odds of post-neonatal mortality were 1.48 times higher among babies born to mothers in Burundi and 1.33 times higher in Uganda compared to the reference country Ethiopia. The possible justification might be due to the difference in socioeconomic status, the health system, and health infrastructure variations.

The main strength of this study is the use of data from an adequately representative sub continental population. This is the first DHS-based study on post-neonatal mortality and factors in East Africa, as far as we are aware. The study's conclusions have a big impact on policy, especially when it comes to figuring out what measures are needed to consistently lower post-neonatal mortality. While our study highlights the influence of geographic disparities in outcomes, we acknowledge that the reliance on secondary DHS data precludes the inclusion of nuanced variables, such as maternal psychological health and cultural practices, which might significantly influence outcomes. Furthermore, we recognize that our analysis does not delve into the health system differences between countries, which could also play a critical role in shaping these disparities.

## Conclusion

According to this study, post-neonatal mortality is higher in developing countries, particularly in East Africa. Post-neonatal mortality was influenced by factors such as earliest gestational ages, multiple pregnancies, low birth weight, lack of formal education, failure to receive tetanus shots before birth, first birth order, and birthplaces in Burundi and Uganda. Therefore, emphasis should be given on children born to mothers in the lowest age group, those born of multiple pregnancies, without formal educations, who did not receive tetanus shots prior to birth, and who were born in the first birth order.

## Data Availability

The datasets presented in this study can be found in online repositories. The names of the repository/repositories and accession number(s) can be found below: http://www.dhsprogram.com.
